# Characteristics and Comorbidities Influencing Mortality Risk Among Hereditary Angioedema Patients

**DOI:** 10.36469/001c.141747

**Published:** 2025-07-17

**Authors:** Subhan Khalid, Alan T. Hitch

**Affiliations:** 1 Data Science Harrisburg University of Science and Technology, Harrisburg, Pennsylvania; 2 Data Science Harrisburg University of Science and Technology

**Keywords:** Bayesian networks, directed acyclic graphs, patient outcomes, hereditary angioedema, epidemiology

## Abstract

**Background:** Patients with hereditary angioedema (HA) face a heightened mortality risk due to multiple factors. **Objective:** The purpose of this study was to identify patient demographics or comorbidities associated with higher mortality risk using Bayesian network analysis. **Methods:** Data from the 2021 Nationwide Inpatient Sample were used to identify hospitalized patients with HA. Patient demographics, comorbidities, and severity measures were analyzed, and a Bayesian network model was developed to assess factors contributing to mortality risk. Structure learning was performed using a directed acyclic graph and probability estimating using Bayesian inference. Model performance was validated using a 70/30 training-testing split and assessed via area under the curve. **Results:** Older HA patients and those with autoimmune conditions, hypertension, or low income were at higher risk of mortality. Elevated risk was also observed across certain racial groups, insurance types, and income levels. Notably, older Black patients from the Midwest exhibited the highest estimated mortality risk. **Conclusion:** The Bayesian network demonstrated strong predictive performance, highlighting its potential for identifying high-risk subgroups and supporting targeted clinical interventions.

## BACKGROUND

Hereditary angioedema (HA) is a rare genetic condition characterized by recurrent swelling episodes in various parts of the body, including the skin, upper airway, and respiratory system.^1^ Symptoms can range from painful swelling of the extremities or face to severe abdominal pain, nausea, vomiting, diarrhea, and life-threatening laryngeal edema.^1^ The underlying mechanism of HA is most often a deficiency or dysfunction in the C1 esterase inhibitor (C1-INH) protein, which helps regulate the complement system. The condition is caused by muttions in the *SERPING1* gene, which encodes the C1-INH protein, and follows an autosomal dominant inheritance pattern that can manifest at any age. Attacks can occur unpredictably, triggered by stress, infections, or trauma, which may worsen symptoms and lead to hospitalization.^2^

HA is classified into 3 types: type I (85% of cases) involves low levels of C1-INH, type II (nearly 15% of cases) involves dysfunctional C1-INH, and type III, the rarest type, involves normal C1-INH levels but mutations in the *F12* gene that affect factor XII.^3^ Blood tests are conducted to assess C1-INH levels, including checking for low C4 levels, which drop during attacks. For a definitive diagnosis, molecular testing is used to detect *SERPING1* mutations. Due to its variable presentation, HA is often underdiagnosed or misdiagnosed, leading to delayed treatment.^4^

Mortality risk in HA has gained attention in recent years, with research focusing on exacerbating factors and fatal outcomes. Mortality risk is significantly elevated during upper airway attacks, especially when treatment is delayed.^5^ Fatal events are primarily linked to laryngeal edema, causing airway obstruction and suffocation.^6^ Individuals with severe attack histories, delayed diagnoses, or inadequate treatment face higher mortality risks.^6^ Comorbidities such as cardiovascular disease and obesity may increase the likelihood of fatal outcomes. Although the overall mortality risk of HA remains low, further studies are needed to understand contributing factors.

Early diagnosis and timely treatment are important to improve quality of life and reduce disease burden.^4,7^ Self-treatment, novel biologics, and prophylactic strategies are essential to effective management, but access to therapies must improve. Further research on mortality risk is essential. Older studies reported mortality rates of 30% to 50% due to laryngeal edema.^8,9^ While effective treatments have since reduced this risk,^10,11^ delayed diagnosis, treatment inaccessibility, and patient comorbidities remain significant risk factors, especially in low-resource settings. Studying mortality risk in HA patients is crucial for identifying high-risk factors, such as demographics and comorbidities; improving medications for preventing attacks; and reducing the likelihood of life-threatening situations.

Bayesian network (BN) models are an ideal tool for predicting mortality risk based on patient demographics and comorbidities due to their ability to model uncertainty and complex dependencies between various factors.^12^ BN models can estimate the relationships between variables of a probabilistic model for a domain^13^ and infer the probability of subsequent events. These models have been used to assess risk factors of cardiovascular disease,^14^ risk prediction,^15^ health status of leukemia patients,^16^ causal relations between food and health,^17^ and relationships between comorbidities and type II diabetes.^18^ A BN framework can be used to calculate conditional probabilities between factors and examine the changes in the probability of risk of mortality, while conditioning on certain factors of interest.^19^ BN models offer better interpretability by visualizing the relationships between variables clearly and understandably, making it easier to communicate results to physicians and policymakers.

Directed acyclic graphs (DAGs) can be used as part of the BN modeling workflow. A DAG provides a visual representation of the statistical dependencies between variables. It represents a sequence of activities in which information moves forward without returning to a previous step or feedback loops.^20^ DAGs help model probabilistic relationships and are commonly used in epidemiology.^21^ For example, Tennant^22^ emphasized the use of DAGs to identify confounders in healthcare studies, Byeon^23^ exhibited the use of DAGs for clinical research and compared them with the traditional approach of *P* values for determining confounding, and Sonis^24^ used DAGs to show effects of posttraumatic stress disorder on Parkinson’s disease. DAGs are acyclic, meaning information can only flow forward, and without feedback loops, which limit the creation of dynamic processes. DAGs assume conditional independence between variables, which may not hold in all real-world situations, leading to potential inaccuracies in the model.^25–27^ The dependencies must align with the assumptions of the data generating process when constructing a DAG. For instance, when suggesting that income affects diabetes, there must be a scientific study showing the association between these two variables.

The objective of this analysis was to use a BN model to ascertain, in patients who have been hospitalized with an HA attack, which demographics or comorbidities put them at a higher mortality risk. These findings may inform physicians as to which patients are at higher risk of mortality and require closer management of their symptoms and conditions.

## METHODS

The Nationwide Inpatient Database (NIS) for 2021 was used for this analysis. The NIS, the largest publicly available inpatient healthcare database in the United States, contains yearly records of inpatient utilization, access, cost, quality, and outcomes from around 7 million hospital stays.^28^

**Figure 1.**
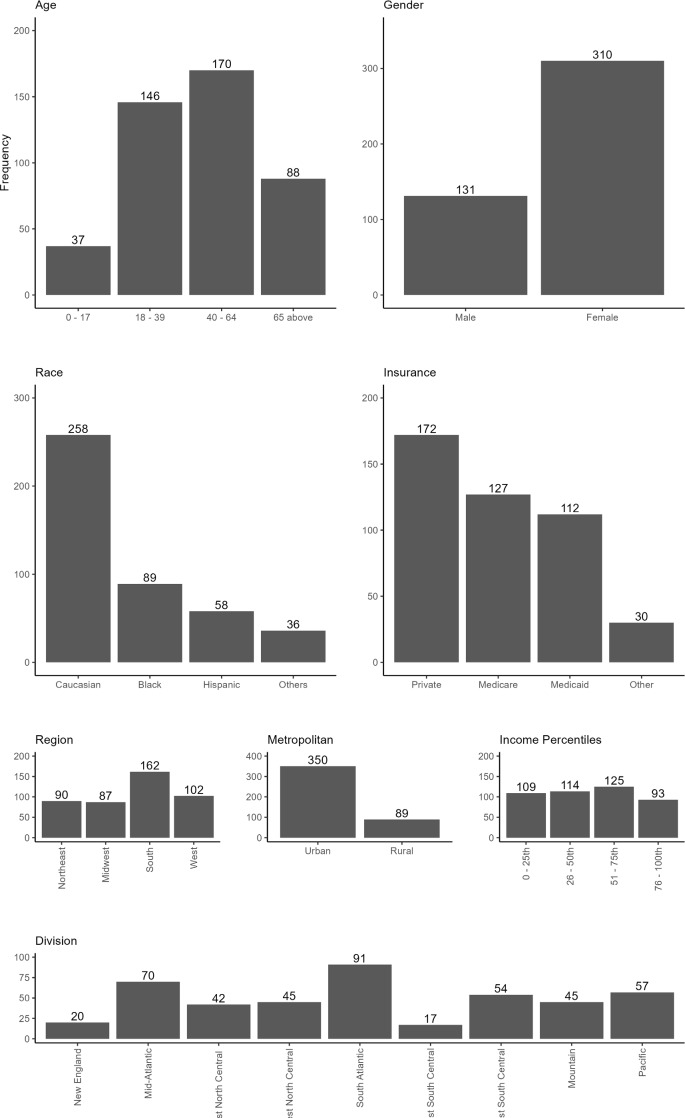
Bar Plots for Demographics for Patients in the HA Cohort (N = 441) Abbreviation: HA, hereditary angioedema. Other race category: Asian, Native American, other and missing. Other insurance category: Self-pay, other sources.

The *International Classification of Diseases, Tenth Revision* (ICD-10) diagnosis code^29^ D84.1 was used to identify patients who experienced an HA attack and were admitted to a hospital. Of 441 patients were assigned to the HA cohort, 70% were female, 58% were White, and 39% were 40 to 64 years of age (**Figure 1**). Private insurance (40%) was most prominently used to cover healthcare expenses. Most of the patients (80%) lived in urbanized areas, 37% were in the South, while 23% were in the West. Patient numbers were similar across income quartiles, slightly higher in the 3rd quartile. The distribution division shows more patients living in the South-Atlantic (20%), Mid-Atlantic (16%), with the fewest in New England and the East South-Central region (4%).

**Figure 2.**
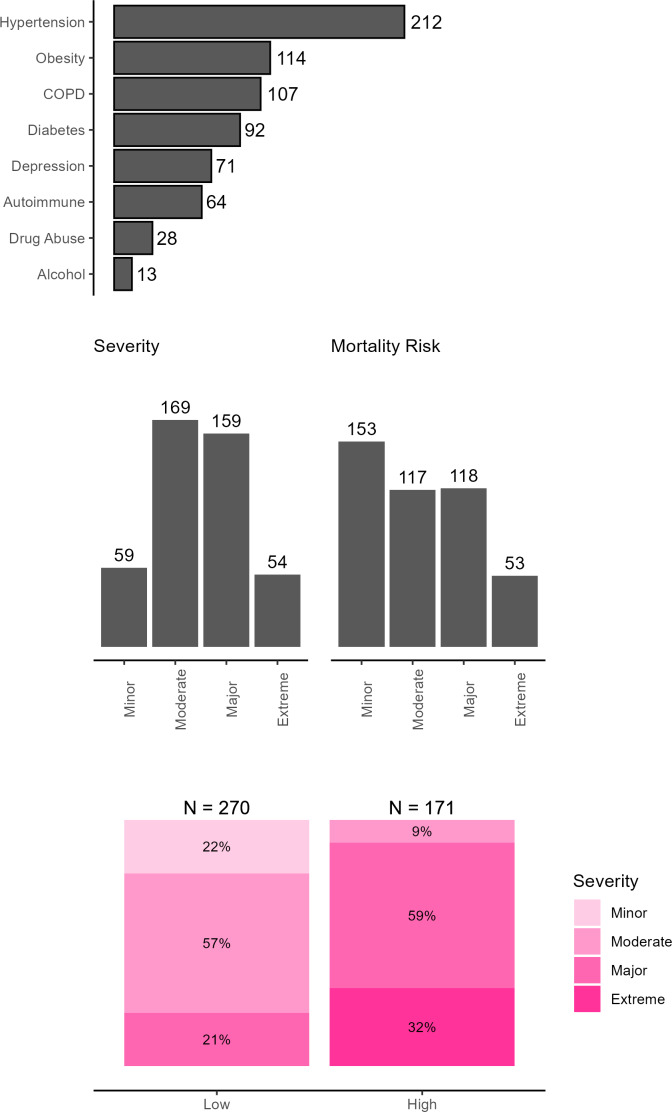
Bar Plots for Comorbidities, Severity, and Mortality Risk for Patients in the HA Cohort (N = 441) Abbreviation: HA, hereditary angioedema.

The most common comorbidity^30,31^ in the sample was hypertension (48%), followed by obesity (25%), chronic obstructive pulmonary disease (24%), and diabetes (21%) (**Figure 2**). These comorbidities were included in the NIS database as binary variables signifying patient comorbidities at time of hospitalization.

Severity of disease and mortality risk at admission were derived in the NIS database using All Patient Refined Diagnosis Related Groups (APR-DRGs) classification system developed by 3M Health.^28^ This classifies patients into 4 categories: minor, moderate, major and extreme (**Figure 2**). Most patients were in the moderate and major categories for both measures. Fewer patients were classified at the extreme ends—either minor or extreme severity. Minor and moderate risk categories were used to create low-risk categories, while major and extreme risk categories were used to create high-risk categories. Almost 38.7% of patients faced a high mortality risk at the time of admission with varying severity.

**Figure 3.**
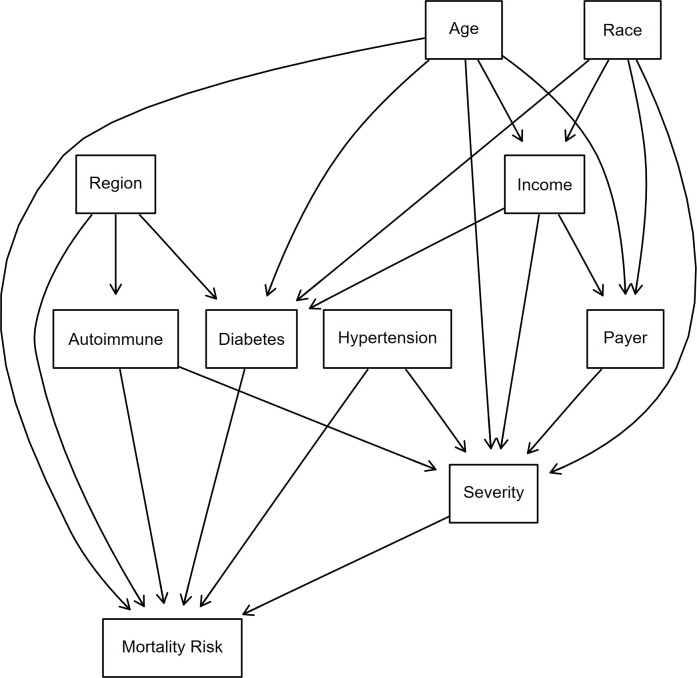
DAG Used for BN Analysis and Interplay Between Patient Demographics and Comorbidities Abbreviations: BN, Bayesian network; DAG, directed acyclic graphs. The direction of the arrows does not imply causation but rather a statistical relationship.

Bayesian structural learning can be divided into 2 phases: structure learning and parameter estimation.^32–34^ For structural learning, the DAG was used (**Figure 3**). The DAG was developed based on a literature review on how demographics and comorbidities intermingle and propagate toward a patient being at higher risk of an HA attack. Nine discrete predictor nodes were included. Uniform priors were assigned to each node using a Dirichlet distribution with ɑ = 1, meaning the probability of any category of a node occurring was equivalent. The Dirichlet distribution is a multivariate generalization of the beta distribution and is used because the parameters of a discrete distribution (the conditional probabilities) must sum to 1.^35–37^ All analyses were conducted in R version 4.4.1 using the packages bnlearn^38^ and Rgraphviz.^39^

Age is an important determinant of mortality risk, especially in HA. Older patients are at higher risk of complications due to age-related bodily changes and the presence of more comorbidities. Older HA patients may experience more frequent or severe disease episodes and may cause difficulties in treatment due to complicated cases.^40^ Certain racial groups may face difficulties in healthcare access and overall disease management^41^ and may affect the HA symptom management. These disparities could lead to patients being susceptible to a higher mortality risk.^42^ Lower-income patients or patients receiving Medicare/Medicaid benefits may face barriers such as the unavailability of specific treatments or the means to follow a treatment regimen consistently. This might cause problems for HA patients in managing their symptoms, as HA treatment and adherence to regimen might be expensive, and difficulties in staying on medication could cause flare-ups or frequent HA attacks. This could contribute to higher mortality risk.

Since the evidence does not support a substantial difference between males and females with regard to HA attacks, sex was not included as a covariate.^43^

Region can affect patients’ lifestyle and access to healthcare, which can affect mortality risk. Geographic differences in healthcare quality and availability of specialists can result in poor health outcomes for patients.^44^ This might affect HA patients due to unavailability of advanced HA treatments and specialized doctors to accurately diagnose and treat patients.

Comorbidities such as hypertension, autoimmune diseases, and diabetes could also put HA patients at higher mortality risk. Hypertension could increase the chances of heart disease,^45^ which can be particularly dangerous for HA patients.^46^ A compounding effect could occur when trying to manage two difficult diseases that require vigilance and complex clinical management. Autoimmune diseases lower immunity and response to treatments^47^ and thereby make managing and treating HA symptoms difficult, as HA patients may not respond to regimens or may face more frequent and severe episodes of HA. Diabetes, another common comorbidity, can negatively affect immune function, making it harder for patients to recover from HA attacks and further increasing mortality risk.^40,48^ The severity variable in the DAG indicates how critical a patient’s condition was at the time of admission. A higher severity at admission would indicate the need for more critical care and a higher likelihood of complications during hospitalization.^49^

While DAGs are easy to visualize and interpret, their structure relies on assumptions that may introduce oversimplifications, which could affect the model’s generalization.^50–52^

The second phase of Bayesian structure learning included parameter estimation, where the conditional probability tables for each node in the network were trained using the data. Prior distributions were assigned to the CPTs of each node before observing the data.^53^ We used the DAG and the observed data as inputs and used Bayesian inference to update the priors, resulting in a posterior CPT distribution for each node.^50^ The posterior distribution balances the prior distribution with the information gained from the data. This allows for parameter estimates incorporating uncertainty, making the Bayesian method less prone to overfitting than maximum likelihood estimation. By incorporating priors into the learning process, Bayesian estimation offers a more robust approach, particularly in cases where the data might be sparse or prior knowledge about the relationships in the model exists.^54^

For learning the network, data were split 70% to 30% for training and testing. This allows the model to understand the patterns in the data through training and then use test data to validate whether the model can be generalized to new data and classify patients into high- or low-risk mortality categories. The area under the curve (AUC) was calculated for both training and testing data to validate the predictions made.

## RESULTS

**Figure 4.**
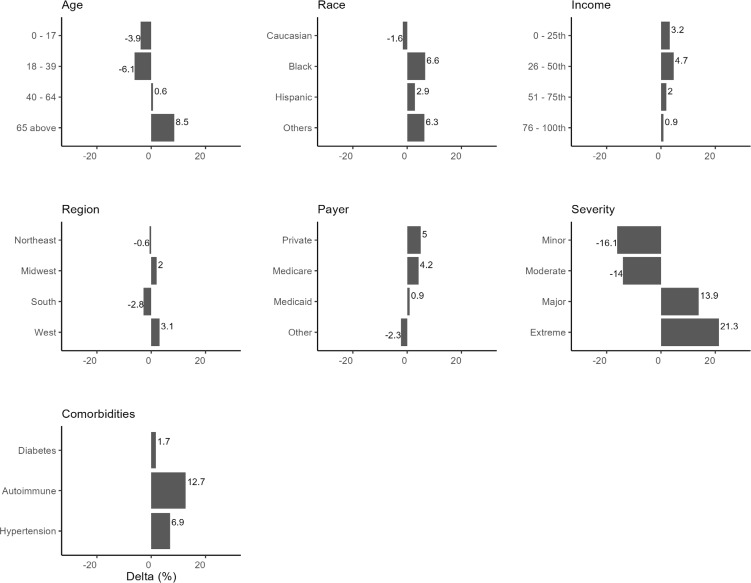
Changes in Estimated Probability of Mortality Risk Conditioning on the Predictor Nodes Abbreviation: AUC, area under the curve.

After applying the DAG to the data, the estimated probability of mortality risk for HA patients was 41.6%. This value represents the model’s posterior probability estimate—not the raw prevalence of high risk in the data (38.8%). All subsequent changes in predicted risk are benchmarked against this estimated baseline (**Figure 4**).

Conditioning on specific demographics/comorbidities reveals their downstream effects on mortality risk (**Figure 4**). Age played a significant role in mortality risk, with younger patients (≤17 years and 18-39 years) experiencing a decrease in mortality risk of 3.9% and 6.1%, respectively, from the baseline mortality risk of 41.6%. In comparison, older patients (≥65 years) showed an 8.5% increase in mortality risk. Racial differences were observed, with Black patients showing a 6.6% increase in mortality risk vs baseline and Hispanics and other racial groups exhibiting increased risks, suggesting that race may be an important factor influencing HA mortality risk.

Compared with the model-estimated baseline risk (41.6%), patients in the first quartile (lowest) income group had a 3.2% increase, compared with 4.7% for the third quartile. Both lower-income quartiles were associated with elevated risk, but the effect was slightly greater for the second-lowest income group. Regional differences also exist, with the South showing a notable decrease in mortality risk of 2.8%. In comparison, the West exhibited an increase of 3.1%, indicating that regional factors such as access to healthcare may play a part in mortality risk.

Private insurance and Medicare beneficiaries show a higher mortality risk by 5% and 4.2%, respectively. Hypertension and autoimmune conditions elevated mortality risks, with hypertension contributing to a 6.9% increase and autoimmune conditions associated with a 12.9% increase, reflecting the known complications associated with these comorbidities. Diabetes increased mortality risk by only 1.7%.

The severity of the patient’s condition at the time of admission was strongly associated with mortality risk. Patients with minor and moderate severity conditions experienced a decreased mortality risk compared with the 16.1% and 14% baseline. In contrast, those with significant and extreme severity showed higher risks, particularly for those classified as extreme, where mortality risk increased by 21.3%.

Bayesian analysis can be used to understand how mortality risk changes conditioning on several nodes. The BN incorporates soft evidence, meaning the observations are treated probabilistically rather than deterministically. The associated probability for the 2 conditioned nodes is not exactly 1, as the evidence is integrated as a distribution over possible values, rather than a hard assignment.^55^ Conditioning on hard evidence might commit too strongly to a specific scenario, whereas soft evidence reflects real-world ambiguity, leading to more conservative and robust predictions. Soft evidence interacts more sensitively with prior probabilities in the network. Because it is a probabilistic input, the final inference depends more on the priors and less on a single piece of “certain” data.

Conditioning on Black patients residing in the Midwest, the mortality risk increased to 50.2% from baseline of 41.6%, suggesting that being both Black and from the Midwest was associated with a much higher mortality risk. If HA patients were Black and from the South, the estimated probability of mortality risk increased to 45.5% from baseline. While this is a lower increase than Black patients from the Midwest, it still suggests that race and region together may contribute to higher mortality risk.

If HA patients were Black, from the Midwest, and aged 65 or above, the estimated mortality risk increased significantly to 59.7%, an increase of 18.5% from the baseline level of 41.6%. This significant increase highlights the compounded effect of age, race, and region on HA mortality risk and suggests that the interaction between racial disparities, regional healthcare access, and the aging process may exacerbate the mortality risk in this patient group. Conditioning on Hispanics using Medicare or Medicaid, the probability of mortality risk increased to 47.9% from the baseline of 41.6%.

**Figure 5.**
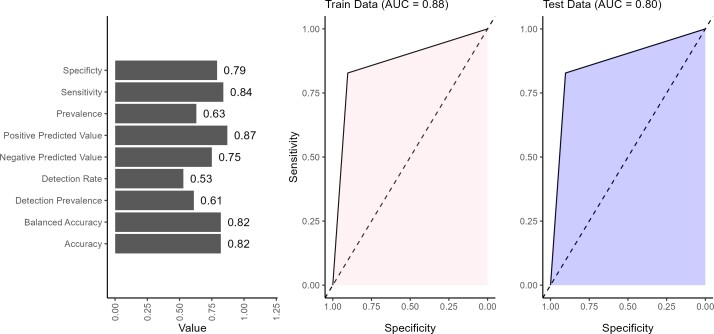
Validating Predictions Made by Bayesian Network Using 30% Test Data

The performance of the BN model showed strong overall results across the validation metrics (**Figure 5**). The model achieved an accuracy of 82%, indicating that the BN network correctly predicts the outcome in 82% of cases. Balanced accuracy (82%) reflected the model’s ability to reasonably predict positive and negative outcomes without favoring one, which is important for maintaining fairness in predictions. The sensitivity of 84% indicated that the model effectively identifies true positives (patients at high risk of mortality), making it particularly valuable in clinical settings where correctly identifying at-risk patients is important. The 79% specificity suggests that the model is generally good at identifying low-risk patients. The positive predictive value of 87% indicates that it is highly likely to be correct when the model predicts a positive outcome (ie, a patient is at high mortality risk). This is an essential strength in clinical decision-making, as false positives can lead to designing healthcare interventions that target the wrong sub-populations. The negative predictive value of 75% indicates that when the model predicts a negative outcome (a patient at low risk), there is only a 25% likelihood that this prediction is incorrect. This finding highlights a potential area for improvement, as improving negative predictive value could reduce the risk of missed diagnoses in patients who are at high risk but were classified as low risk. The detection rate indicates that 53% of all individuals in the data set were correctly identified by the model as having the outcome of interest. In other words, just over half of the total population were true positive. The detection prevalence revealed that 61% of all predictions made by the model were classified as positive, regardless of whether those predictions were correct. This suggests that the model tends to predict the positive class more frequently. The BN performed well in classifying low or high mortality risk, with notable strengths in sensitivity, positive predictive value, and balanced accuracy. However, refinements in specificity, negative predictive value, and detection could further enhance its reliability in clinical decision-making.

The AUC values of 0.88 on the training data and 0.80 on the test data suggested that the model performs better on the data it was trained on than the unseen test data (**Figure 5**). The BN only provided prediction in discrete form; hence, the AUC curves were straight lines. An AUC of 0.88 on the training set indicated that the model effectively distinguished between high and low risk in the training data, showing strong performance in identifying patterns and relationships within this data set. However, the drop to 0.80 on the test data indicated a slight reduction in performance when the model is applied to new, unseen data.

## DISCUSSION

Age is an important factor in mortality risk, with older patients often experiencing increased vulnerability due to physiological decline, comorbidities, and challenges in disease management. In this study, older patients exhibited a higher mortality risk of HA. Advanced age is often associated with physiological decline, making older HA patients more susceptible to severe and frequent attacks.^56^ Aging is linked to an increased burden of comorbidities and impaired immune function, which can complicate HA treatment and management.^57^ Older patients may also face delays in diagnosis or mismanagement due to overlapping symptoms with other age-related conditions.^58^ These factors highlight the need for tailored healthcare approaches for elderly patients and ensuring timely and effective interventions to reduce mortality risk.

Socioeconomic factors, including income level, insurance type, and race, played a role in mortality risk among HA patients. Patients in the second lowest income quartile (45.9%) had a slightly higher estimated mortality risk than the baseline probability of 41.6%. While it was initially hypothesized that the lowest income group (first quartile) would face the highest risk, the model estimated the greatest increase in predicted mortality for the second quartile. This indicates that the two lowest-income quartiles were associated with elevated risk, but the effect was slightly greater for the second-lowest income group. Lower-income patients may face barriers such as the unavailability of specific treatments or the means to follow a treatment regimen consistently.^59^ This might cause problems for HA patients in managing their symptoms, as HA treatment and adherence to a regimen might be expensive, and difficulties in staying on medication could cause frequent HA attacks or flaring up of symptoms. Patients insured by Medicare (45.4%) experienced a higher probability of mortality risk than those with private insurance (44.3%). Medicare is generally accepted in limited number by healthcare providers,^60^ which might delay healthcare provision for these patients. Medicare and Medicaid are often associated with older or low-income populations who may face barriers to timely and effective health care, which could contribute to poorer health outcomes and higher mortality risk. Additionally, being Hispanic and on Medicare was associated with a higher mortality risk (47.9%). This may reflect other social determinants of health, such as language barriers, access to healthcare, and socioeconomic challenges, which could further increase the risk. The combined effect of racial disparities and Medicare or Medicaid has been documented in other disease areas,^61,62^ suggesting that these patients may be especially vulnerable to HA attacks and need monitoring and vigilance when controlling their symptoms. The effect of income, payer type combined with race, reflects difficulties in healthcare access, obtaining HA medication, staying persistent on treatment regimen and preventing the onset of HA attacks.

Geographical and disparities among other races also play a role in influencing HA mortality risk, highlighting the impact of healthcare access, provider expertise, and socioeconomic factors on patient outcomes. Black patients (47.8%) and those of other races (48.7%) were at higher mortality risk than White patients (39.6%). Certain racial groups may face difficulties in healthcare access and overall disease management.^63^ In the context of HA, racial differences may affect the management of HA symptoms, and if the disease becomes more complicated, then so does the response to treatment. These disparities could lead to a higher risk of mortality. Patients in the Midwest (43.2%) and the West (44.7%) had a slightly higher estimated mortality risk relative to the baseline level of 41.6%. Geographic differences in healthcare quality and availability of specialists can result in poor health outcomes for patients.^64^ The Midwest could specifically be associated with a lack of physician expertise in controlling HA attacks or treating HA patients.^65,66^ Although the risk of mortality is still increased in the South, the increase was much higher in the Midwest. This disparity requires further investigation and potentially improving the HA treatment options available in those hospitals. Socioeconomic factors and lifestyle could also play a role that could disproportionately affect Black HA patients in different regions. Improving healthcare infrastructure and lifestyle is needed to improve outcomes for HA patients in these regions.

Patients with preexisting comorbidities face a higher risk of mortality, as they can complicate the management of HA and exacerbate overall health outcomes. Comorbidities such as autoimmune diseases (53.9%) and hypertension (48.1%) appeared to substantially increase the likelihood of mortality risk compared with the baseline level of 41.6%. This finding aligns with medical literature for autoimmune diseases, which can lead to severe complications, including organ damage, chronic inflammation, and increased susceptibility to infections.^67^ Hypertension could increase the chances of heart disease, which can be particularly dangerous for HA patients.^57^ The results also show a small effect of diabetes on HA mortality risk (1.7% increase). Although diabetes had been hypothesized to have a higher effect, the literature supports that HA mortality is more related to the acute management of angioedema episodes rather than diabetes.^68^ The evidence underscores that higher HA mortality risk exists for patients suffering from chronic comorbidities such as autoimmune diseases and hypertension, and less for diabetic patients.

The severity of a patient’s condition at the time of hospitalization plays a critical role in determining mortality risk. Disease severity at the time of hospital admission showed considerable variation, with individuals suffering from major (55.1%) or extreme (62.5%) severity having a substantially higher probability of mortality risk. This finding highlights the importance of severity in determining the mortality risk^69–71^ and sheds light on how patients presenting with severe HA attacks require urgent, appropriate assistance to manage their HA symptoms.

The findings from this study contribute to advancement of hereditary angioedema treatments, addressing the need for specific medications for higher risk groups. It also contributes to the need for improved prophylactic medication for older, hypertensive and autoimmune compromised patients to manage HA symptoms and reduce chances of fatality.

### Limitations

It is important to acknowledge that the DAG used for this study might not account for literature that shows opposite relationships or cycles between nodes (eg, diabetes could take a toll on a person’s health and affect their earning potential.) The assumption that only one pathway is from one node to another might not hold in certain situations.

While studies have utilized the 3M-derived mortality risk,^72,73^ it is acknowledged that using it introduces some uncertainty regarding the degree of overlap between predictors and the outcome. Thus, caution is warranted in interpreting the model as offering entirely independent insights. Future work should also incorporate externally validated mortality data to test the model’s generalizability and predictive utility more rigorously.

While the 3M algorithm is proprietary and thus not fully transparent, it is derived from primary diagnosis, lab results, diagnoses (ICD-10-CM), procedures (ICD-10-PCS), patient age, and comorbidities.^28^ The precise weights used in the derivation are unknown; however, using mortality risk as the outcome variable in this analysis raises the possibility of circular reasoning and introduces some uncertainty regarding the degree of overlap between predictors and the outcome. Although this may affect interpretation and model novelty, the aim is to quantify interdependence between patient characteristics and comorbidities and understand the specific probabilistic changes in mortality risk when conditioned on patient characteristics and comorbidities.

With a sample size of 441, certain combinations of parent variables may result in small or sparse subgroups. This study used parameter smoothing, which applies Dirichlet smoothing for parameter estimation to prevent zero-probability issues in low-count cells. The model showed that among patients predicted to be at low risk of mortality, 25% were actually at high risk. A model used to rule out high-risk patients would ideally require a much higher NPV to ensure patient safety. While the model may assist in risk stratification and prioritization, it should not be used as a standalone tool to exclude patients from further monitoring or intervention. Future work may improve negative predictive value by including more covariates.

## CONCLUSION

These findings provide valuable insights into the factors influencing mortality risk for HA patients, with BN analysis offering a detailed understanding of complex dependencies among patient demographics and comorbidities. These results have ramifications for both patients and physicians to improve HA symptom management and preventing onset of life-threatening situations.

Older HA patients, Medicare beneficiaries, or patients with autoimmune conditions, hypertension, or low income are at higher risk of mortality. Understanding this aids physicians in managing HA symptoms for these higher-risk patients to prevent HA attacks and onset of life-threatening situations. The findings from this study contribute to advancement of HA treatments, addressing the need for specific medications for higher-risk groups. It also contributes to the need for improved prophylactic medication for older, hypertensive, and immunocompromised patients to manage HA symptoms and reduce likelihood of fatality.

### Disclosures

The authors declare no conflicts of interest and received no external funding for this research. All analyses and writing were completed independently without additional assistance.

## Data Availability

To support reproducibility and transparency, author codes are available on request. All variables were taken from the NIS database as-is. The NIS 2021 database can be used to apply the codes and replicate results; however, patient-level data cannot be shared due to privacy legislation.
